# Sexual Dysfunction and Infertility in Male Patients With Spinal Cord Injury: A Narrative Review

**DOI:** 10.7759/cureus.111929

**Published:** 2026-07-01

**Authors:** Gerasimos Katopodis, George Bablekos, Elias Vasiliadis, John Vlamis, Aikaterini Kotroni, Spyridon G Pneumatikos

**Affiliations:** 1 Physical Medicine and Rehabilitation, KAT Hospital, Athens, GRC; 2 Occupational Therapy, School of Health Sciences and Caring, University of West Attica, Athens, GRC; 3 3rd Department of Orthopaedic Surgery, School of Medicine, National and Kapodistrian University of Athens, KAT Hospital, Athens, GRC; 4 Physical Rehabilitation Medicine, KAT Hospital, Athens, GRC

**Keywords:** infertility, male, sexual dysfunction - physiological, sexual dysfunctions - psychological, spinal cord injuries

## Abstract

Spinal cord injury (SCI) is a life-altering neurological condition that profoundly impairs male sexual function, fertility potential, and overall quality of life (QoL), affecting multiple aspects of physical, psychological, and social well-being. These effects arise from a complex interplay of neurological disruption, autonomic dysfunction, endocrine alterations, and psychosocial factors, with the level and completeness of injury serving as key determinants of functional outcomes. In recent years, growing evidence has highlighted the underlying neurophysiological mechanisms of sexual dysfunction, as well as significant impairments in semen quality primarily related to reduced motility, oxidative stress, and sperm DNA damage. Alongside these findings, advances in therapeutic approaches - including pharmacological treatments, assisted ejaculation techniques, and assisted reproductive technologies - have improved the management of sexual and reproductive dysfunction in men with SCI. This review aimed to synthesize evidence on sexual dysfunction, fertility impairment, semen quality, and therapeutic management in adult men with spinal cord injury, while also addressing the need for structured approaches to optimize patient care.

## Introduction and background

Spinal cord injury (SCI), of traumatic or non-traumatic origin, has a global incidence of 40-80 cases per million population and is associated with significant long-term complications requiring lifelong adaptation [1]. According to data from the World Health Organization (WHO), approximately 70% of SCIs are traumatic, with men accounting for the majority of the patient population (79%) and occurring at a mean age of 42 years, indicating that a substantial proportion of affected individuals are of reproductive age [1]. Notably, up to 75% of men with SCI experience sexual dysfunction and infertility due to disruptions in sensory, motor, and autonomic pathways involved in arousal, erection, and ejaculation [1,2].

Erectile function is primarily mediated by the autonomic nervous system, with parasympathetic activation (S2-S4) facilitating penile tumescence and sympathetic pathways (T11-L1) exerting inhibitory control [3-6]. Furthermore, motor neurons involved in ejaculation are located in Onuf’s nucleus within the sacral spinal cord and are projected via the pudendal nerve to the pelvic floor muscles, including the bulbospongiosus, ischiocavernosus, and external urethral sphincter muscles [3-6]. The role of the nitric oxide (NO) pathway in erectile dysfunction is underlined in the relevant literature [3]. Efferent signals are transmitted to the corpora cavernosa via long preganglionic pelvic nerves and short postganglionic cavernous nerves, also known as non-adrenergic, non-cholinergic (NANC) fibers. Nitric oxide, released by NANC fibers, plays a central role in the erectile function [4]. Sensory input is conveyed via the dorsal penile nerve through the pudendal pathway to the lumbosacral spinal cord, while psychogenic stimuli may activate either sacral or thoracolumbar pathways [3,4].

As for the erectile function, it is important to note that diabetic and non-diabetic neuropathies, as well as spinal cord injury and pelvic surgery, cause the appearance of neurogenic erectile dysfunction due to disruption of peripheral or central pathways [7]. At this point, the usefulness of the application of the P-LI-SS-I-T-based algorithm model (Permission, Limited Information, Specific Suggestions, Intensive Therapy) in the treatment of sexual dysfunction of neurogenic etiology should be reported [8].

Also, in men, waist circumference measurement appears to be associated with the imminent onset of erectile dysfunction, which may mask the development of systemic atherosclerosis, so cardiovascular risk assessment is required. Furthermore, clinicians should keep in mind that erectile dysfunction precedes the occurrence of cardiovascular disease by an average of two to five years, which is very important in implementing preventive treatment [7]. It should be further reported that sexual function (including parameters such as libido, erection, ejaculation, and orgasm), except for traumatic etiology like traffic accidents and falls, can also be affected by causes like herniated intervertebral discs that injure the spinal cord. It is of utmost importance for men with SCI to realize what they are able to gain in order to improve their sexuality, intimacy, and reproductive health after the injury, with all the means provided to them for their rehabilitation [9].

Ejaculation, also regulated by the autonomic nervous system, consists of emission and expulsion phases, predominantly mediated by sympathetic activity and coordinated contraction of pelvic floor musculature [3-6].

The level and completeness of SCI critically determine sexual function outcomes [4]. Lesions above T10 generally preserve reflexogenic erections but impair psychogenic responses and have greater sexual potential, whereas sacral lesions may preserve psychogenic erections while disrupting reflexogenic erections. In addition, lesions at the thoracolumbar level (T10-L2) may preserve reflexogenic erections but usually result in the loss of emission and ejaculation [4,5]. Ejaculatory dysfunction is common and may include retrograde ejaculation due to sphincter or bladder neck dysfunction, typically associated with lower-level lesions [5].

The IIEF-5 (International Index of Erectile Function - five items) is a short, self-administered questionnaire primarily preferred for the assessment of the severity of erectile dysfunction (ED), focusing on confidence in obtaining an erection, maintaining it during sexual intercourse, and overall ability and satisfaction over the past six months, with scores ranging from five to 25, indicating severity ranging from severe (5-7) to no sexual dysfunction (22-25) [10]. Moreover, it should also be mentioned that in order to clinically evaluate patients with SCI, the American Spinal Injury Association (ASIA) Impairment Scale (AIS), which is a standardized neurological examination to classify the severity of the specific injury, is used [11].

The question being addressed in this narrative review is the thorough investigation and ways to treat sexual dysfunction and fertility disorders, with all the consequences they bring in everyday life and its quality, in men of reproductive age who have suffered a spinal cord injury.

## Review

Materials and methods

For the writing of this narrative review, the Medical Subject Headings (MeSH) terms and keywords that were used were as follows: “Spinal Cord Injuries,” “Infertility, Male,” “Sexual Dysfunction, Physiological”, and “Sexual Dysfunctions, Psychological”. Three separate literature searches were conducted in the Hellenic National Documentation Center by an information specialist, under the supervision of the primary investigator. The search period was from January 1, 2005 to June 30, 2025. The date on which searches were performed was June 30, 2025. The PubMed database was used, and a total of 545 records were initially identified, although there is a risk of selection bias for the use of a single database for literature search. The aforementioned three separate literature searches are described below.

(i) In the first literature search, the combination of the keywords was as follows: “Spinal Cord Injuries” [Majr] AND “Infertility, Male” [Majr]. One hundred articles were found, without any filtering to be applied. (ii) In the second literature search, the combined keywords were as follows: “Spinal Cord Injuries” [Majr] AND “Sexual Dysfunction, Physiological” [Majr]. As a result, 276 articles were found. Then, a first filtering was applied by using the term “Male,” and the number of the identified articles was limited to 243. A second filtering was applied by using the term “/etiology” included in the following combination: “Spinal Cord Injuries” [Majr] AND “Sexual Dysfunction, Physiological / etiology” [Majr], which further reduced the number of articles to 126. Thereafter, a third filtering was performed by using the term “Male”, and the number of articles found was limited to 108. Therefore, from the second literature search, 108 articles were identified. (iii) In the third literature search, the keywords were combined as follows: “Spinal Cord Injuries” [Majr] AND “Sexual Dysfunctions, Psychological” [Majr], and 169 articles were found. Then, a first filtering was applied by using the term “Male”, and the number of articles that were identified was limited to 157. A second filtering was applied by using the term “/etiology” included in the following combination: “Spinal Cord Injuries” [Majr] AND “Sexual Dysfunctions, Psychological / etiology” [Majr], which further reduced the number of articles to 77. A third filtering was applied by using the term “Male”, and the number of articles found was 71.

A total of 279 articles were ultimately identified out of the 545, after all three searches, and 266 articles were excluded. Also, out of the 279 remaining articles, 78 articles were removed as duplicate records, and 201 articles were considered to be potentially eligible for retrieval. Although the article is a narrative review, titles, abstracts, and full texts were screened by two co-authors acting independently of one another, while, upon completion of the screening, an inter-reviewer consensus was also implemented. Moreover, non-English studies, studies involving geographically isolated populations, studies that did not refer to humans, as well as studies that did not meet the search period criterion, were all considered as limitations in the literature search. Additionally, out of the remaining 201 articles, 104 articles were excluded because they did not meet the above criteria. As a result, 97 articles were considered eligible according to their title, abstract, and full-text content.

Out of these, 33 articles were further excluded due to the following reasons: (i) 15 studies because they did not have sufficient correlation with the question being addressed in the article, (ii) six studies because they had limited sample size, (iii) five studies because the therapeutic interventions that were proposed had controversial effectiveness, (iv) five case studies due to lack of a control group and limited generalizability of the results, (v) two studies as preliminary data, because although they were found as online preprints forms, they were not yet formally published. Also, following the primary literature search, nine additional, more recent studies from PubMed, Scopus, Google Scholar, and National Library of Medicine databases were identified through manual screening of references and citation tracking, and they were included in the article as fully compatible with the topic under investigation.

**Figure 1:**
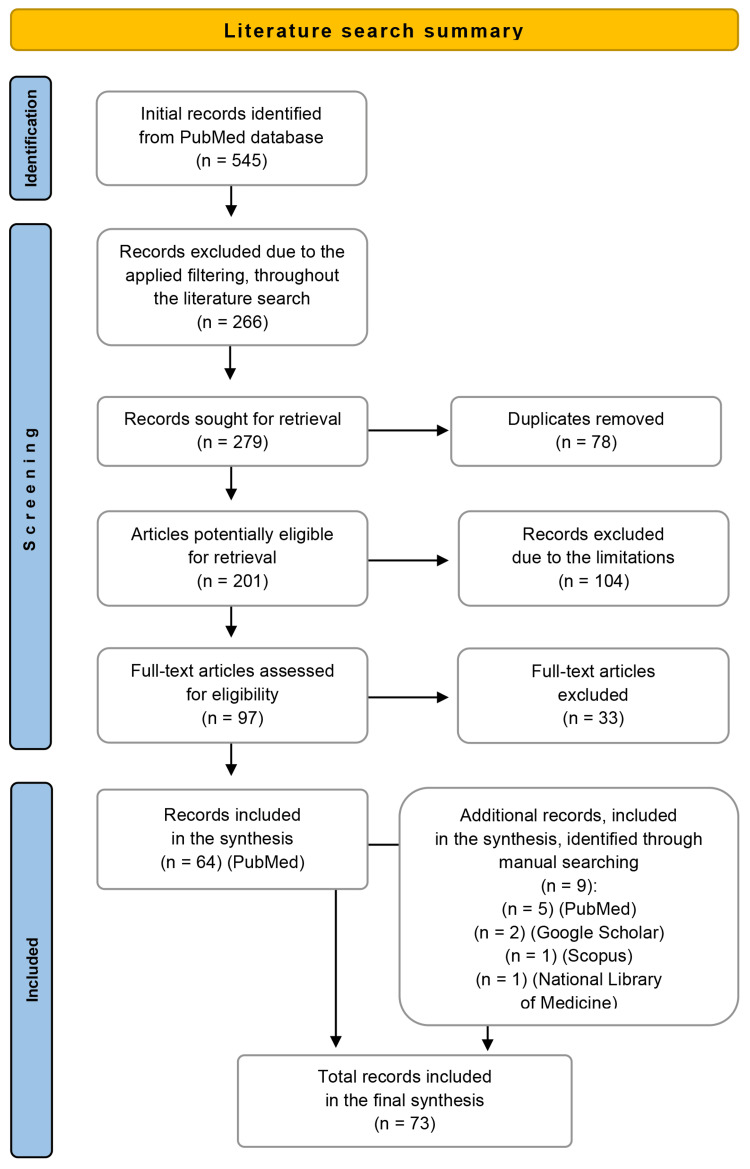
Literature search summary

Thus, 73 studies were included in the final analysis. The literature search summary is presented in Figure 1. Data on the identity of the studies, as well as main findings and characteristics of studies included in the writing of the manuscript, are presented in Tables 1-4.

**Table 1:** Main findings and characteristics of the studies, based on epidemiology, pathophysiology, assessment of sexual function, and neurophysiology for men with spinal cord injury PLISSIT: Permission, Limited Information, Specific Suggestions, Intensive Therapy; SCI: spinal cord injury

Study	Study design	Sample size (participants)	Level of evidence	Main findings and characteristics
Epidemiology				
WHO [1]	International Collaborative Report and Epidemiological Review	n/a	n/a	Men account for 79% of the total 70% of SCI, with an average age of onset at 42 years of age.
Spinal Cord Injury (SCI) [2]	Epidemiological Data Sheet and Statistical Reference Report	32,000 participants	4	In men with SCI, sexual dysfunction along with infertility occurs in 75%.
Pathophysiology				
Dean and Lue [3]	Narrative Literature Review Article	n/a	5	The study focuses on the role of the nitric oxide (NO) pathway in pathophy­siology and therapy of erectile dysfunction.
Everaert et al. [4]	Narrative Review Article	n/a	4	Erection is found to be cholinergic and nitric oxide (NO)-mediated, while ejaculation function is mainly adrenergic and somatic.
Courtois and Charvier [5]	Narrative Literature Review Article	n/a	5	In men with SCI, sexual dysfunction affects erection, ejaculation, and orgasm, while it also brings about genital congestion. Complete SCI significantly increases the degree of dysfunction.
Crocetto et al. [7]	Narrative Literature Review Article	n/a	5	Medication, surgery, and psychosocial approaches are the main pillars in the modern treatment of erectile dysfunction. Phosphodiesterase-5 inhibitors are the first-line medication to be administered, while penile prosthesis implantation is also a promising surgical treatment. Psychotherapeutic approach should be mainly oriented towards urologic care, specifically in younger patients, as well as in cases of psychogenic erectile dysfunction.
Bershadsky et al. [8]	Observational, Protocol-Implementation study	10 participants (6 men, 4 women)	4 to 5 (based on expert consensus, observational, and program implementation data)	The importance of the adapted PLISSIT-based algorithm for early diagnosis and treatment of sexual dysfunction is underlined. Parameters included in the protocol were functional evaluation, educational consulting, environmental adaptation, as well as structured self- assessment, particularly following the first sexual attempt. The purpose of implementing this specific protocol is to strengthen the targeted multifactorial therapeutic approach often required in these cases.
Zizzo et al. [9]	Narrative Literature Review Article	n/a	4	Parameters such as libido, achievement and maintenance of erection, ejaculation, and orgasm can be impaired after SCI. The awareness and acceptance of the present situation after a spinal cord injury seem to be of great importance for components such as sexual function and reproductive health.
Assessment of sexual function				
Alexander et al. [10]	Systematic Literature Review and Critical Evaluation	n/a	4	The International Index of Erectile Function (IIEF) was used to assess sexual function after SCI. The examination of the ejaculatory capacity and semen analysis was also recommended in order to evaluate male fertility potential.
Gao et al. [12]	Retro­spective Cohort Study	117 male patients	4	It seems that in 70% of men having thoracolumbar fracture combined with incomplete SCI, a significant recovery of their sexual function was observed two years postoperatively.
Neurophysiology				
Rahmani et al. [13]	A Systematic Scoping Review	n/a	4	In patients with SCI, it seems that parameters such as the completeness and the level of injury, the anatomical factor, the social factors associated with the economic status, psychological and social factors, as well as the type of relationship, affect both sexual health and quality of life in these patients.
Chéhensse et al. [14]	Systematic Review and Meta-Analysis Article	3851 participants	1	It seems that in men, there is spinal control of ejaculation via a hypothetical spinal generator of ejaculation (SGE) being located in the L3, L4, and L5 segments.
Virseda-Chamorro et al. [15]	Observational Case-Control Study	98 men with SCI, and 89 men as control group	3b	In men with SCI, the innervation pathways of sexual function were affected. Significant differences were identified concerning electromyographic findings of the bulbocavernosus muscle, pudendal sensory thresholds, somatosensory and genital sympathetic potentials, as well as regarding electromyographic findings of the cavernous bodies following prostaglandin E1 (PGE1) injection.
Tas et al. [16]	Clinical, Cross-sectional Study	17 men, 8 women	4	In SCI patients, bulbocavernosus reflex (BCR), pudendal somatosensorial evoked potentials (pSEP) and perineal sympathetic skin responses (pSSR) were used to assess the remaining sexual function.

**Table 2:** Main findings and characteristics of the studies, based on quality of life (QoL), semen parameters, and ejaculatory dysfunction, for men with SCI SCI: spinal cord injury

Study	Study design	Sample size (participants)	Level of evidence	Main findings and characteristics
Quality of Life (QoL)				
D'Andrea et al. [17]	Multicenter Cross-Sectional Study.	100 consecutive men with SCI	4	In men with chronic SCI, low levels of testo­sterone, including the calculated level of free testosterone, and the degree of impairment for both bowel and bladder function seem to have an important independent role in erectile dys­function accompanied by life dissatisfaction.
Khak et al. [18]	Cross-Sectional Study	37 male veterans with SCI	4	In men with SCI, sleep deprivation and hypertension were found to be significantly related to the emergence of erectile dysfunction.
Taylan et al. [19]	Des­criptive and Cross-Sectional Study	131 married individuals with SCI	4	Sexual self-consciousness and adjustment between men with SCI and their spouses significantly diminished sexual embarrass­ment by simultaneously being considered as an important parameter to prevent further development of sexual dysfunction.
Lai et al. [20]	Cross-Sectional, Mixed-Methods Study	20 men with chronic SCI	4	In men with SCI, sex hormone concentrations, metabolic biomarkers, body composition, and levels of habitual physical activity were examined in an attempt to clarify sexual function. The role of adaptability was of major importance to ameliorate sexual satisfaction, while sexual activity was correlated with physical activity.
Lim et al. [21]	Narrative Review Article	n/a	5	In men with SCI, long-term exercise accompanied by the appropriate diet and supplementary medications seems to positively improve the changes that occur in the human body, thus also resulting in improved sexual function.
Semen Parameters and Ejaculatory Dysfunction				
Chalas et al. [22]	Prospe­ctive, Longitudinal Pilot Study	35 men with SCI	4	In men with SCI, no significant deterioration was found over time regarding semen quality, even if genital inflammation is chronic.
Cito et al. [23]	Retro­spective, Monocenter Case-Control Study	53 couples in the SCI group, and 140 couples in the non-SCI group	3	Between men with SCI and men with idiopathic infertility, no significant differences were found concerning pregnancy, miscarriage, and live birth rates.
Vargas-Banquero et al. [24]	Retro­spective Des­criptive Study	27 men with SCI	3	Sperm concentration was higher in men with cervical cord injuries, while sperma­tozoa progressive mobility was lower in men with complete compared to incomple­te SCI. The elevated sperm DNA fragmentation (SDF) observed in SCI male patients, kept up with leukocytospermia levels and levels of reactive oxygen species (ROS).
Alexandrino et al. [25]	Case-Control Study	24 men with SCI, and 24 controls	3	Men with SCI presented significantly lower seminal zinc concentrations compared to healthy participants in the study.
Qiu et al. [26]	Observational Clinical Study	26 men with SCI, and 16 donors	2b	The quality of semen was significantly poorer in men with SCI compared to healthy men, concerning morphology and sperm motility, hypo-osmotic swelling test (HOS)/eosin staining, sperm DNA fragmentation (SDF), and sperm chromosomal aneuploidies.
Patki et al. [27]	Cross-Sectional, Observational Study	n/a	4	In men with SCI, the occurrence of astheno­zoospermia, which mainly represents low sperm motility and vitality, is promoted. Also, seminal plasma constituents, type of bladder management, and neurogenic impairment of the ejaculatory function appear to be parameters associated with semen quality.
Brackett et al. [28]	Controlled, Cross-Sectional Observational Study	10 men with SCI, 12 men were control group	2	In men with SCI, the sperm DNA fragmentation (SDF) index was significantly greater in their semen compared to controls, although this finding does not seem to be related to anejaculation, necrospermia, or leukocytospermia.
Hamid et al. [29]	Prospective Rando­mized Controlled Trial	74 men with SCI	1b	Repeated ejaculation seems to improve semen characteristics for parameters such as morphology, forward progression, and motility in men with SCI. In these patients, repeated ejaculation is recommended for at least three months prior to attempts at intravaginal or intrauterine insemination.
Soler et al. [30]	Retro­spective Obser­vational Clinical Study	33 men with SCI	4	In men with SCI, the issue of assisted reproduction by using sperm that remains in the prostatic urethra because of ejaculation dyssynergia is examined, within the scope to investigate the possibility of fertilization.
Courtois and Charvier [31]	Retrospective Study	34 men with SCI	4	In men with SCI, premature ejaculation (PE) is associated with L5-S4 lesions. Sacral inhibition during the ejaculation process or simultaneously occurring activation of psychogenic erection are considered to be mechanisms promoting PE in these patients. Lesions can be located in the cauda equina, epiconal, and conus terminalis. Sacral parasympathetic and somatic inhibitory pathways are supposed to be disrupted, thus bearing the imbalance between spinal regulation of ejaculation and brain control.

**Table 3:** Main findings and characteristics of the studies, based on medication for erectile dysfunction and assisted ejaculation techniques, for men with SCI SCI: spinal cord injury

Study	Study design	Sample size (participants)	Level of evidence	Main findings and characteristics
Medication for Erectile Dysfunction				
Tienforti et al. [32]	Systematic Review and Network Meta-analysis (NMA)	1492 men with SCI	1a	In men with SCI, tadalafil medication seems to be the first choice of treatment, among phosphodiesterase type-5 (PDE-5i) inhibitors, in order to manage erectile dysfunction (ED).
Ohl et al. [33]	Rando­mized Controlled Trial	248 men with SCI	1b	In men with SCI, sildenafil constitutes a well-tolerated and effective medication to treat sexual dysfunction. Significant improvement was observed in erections, ejaculation frequency, and intercourse.
Jia et al. [34]	Systematic Review and Meta-Analysis	963 men with SCI	1a	In men with SCI, phosphodiesterase-5 inhibitors (PDE5) constitute the first choice of medication in order to manage erectile dysfunction.
Rizio et al. [35]	Systematic Literature Review	713 men with SCI	3	In men with SCI, sildenafil, tadalafil, and vardenafil constitute medications administered to manage erectile dysfunction. No statistically significant differences in their effectiveness were observed, although tadalafil showed a longer time duration. New medications, like avanafil, are under investigation.
Lombardi et al. [36]	Obser­vational Retro­spective Longi­tudinal Cohort Study (clinical study)	113 men with SCI	4	In men with SCI, sildenafil is considered to be an effective and safe long-term medication to treat erectile dysfunction. Also, erection quality along with sexual satisfaction were found to be significantly ameliorated, following sildenafil administration, in SCI located higher than the T12 level, as well as in incomplete SCI.
Soler et al. [37]	Non-Randomized, Open-label Prospective Trial	240 men with SCI	4	In men with SCI, although sildenafil, vardenafil, and tadalafil were all found to be effective and well-tolerated to manage erectile dysfunction, sildenafil was shown to be more adequate. Lesions located in the upper motor neuron showed better responsiveness to treatment compared to those located in the lower motor neuron and cauda equina.
Guiliano et al. [38]	Rando­mized Controlled Trial	418 men with SCI	1b	In men with erectile dysfunction due to SCI, treatment with vardenafil, for a duration of over than 12 weeks, significantly ameliorated mean per-patient penetration, maintenance, and ejaculation success rates. Flushing, nasal congestion, headache, and dyspepsia were observed as the most frequently occurred side-effects.
Assisted Ejaculation Techniques				
Ibrahim [39]	Video Demon­stration and Clinical Instru­ctional Article	n/a	4 (Case Series) to 5 (Expert Opinion or Case Report)	In men with SCI, penile vibratory stimulation (PVS) constitutes a safe technique for ejaculatory dysfunction. This method was found to be successful with no complications in 86% of patients to whom the level of injury was T10 or rostral.
Fenstermaker et al. [40]	Clinical Review Article	n/a	4	In men with SCI, ejaculatory dysfunction is manipulated via penile vibratory stimulation (PVS) or electroejaculation (EEJ), showing high success rates.
Kasum et al. [41]	Narrative Review Article	n/a	3 to 4 (as a review article rather than a primary clinical trial)	In men with SCI, phosphodiesterase-5 inhibitors, intracavernosal injections, vacuum devices, and penile prostheses are used to treat erectile dysfunction. Penile vibratory stimulation (PVS), electro ejaculation (EEJ), and prostate massage, or surgical sperm retrieval technique, may lead to fatherhood.
Soeterik et al. [42]	Retro­spective Study (Retrospective chart review/case series)	47 men with SCI	4	In men with SCI, it seems that electroejaculation (EEJ) constitutes a highly efficacious method to collect sperm. It has been found that in 227 out of 230 electroejaculations (98.7%), an ejaculate could be obtained. Semen can be collected via EEJ in order to be used in assisted reproductive technologies, with repeated EEJ recommended in case of failure of the first attempt.
Sinha et al. [43]	Narrative Clinical Review Article	More than 1000 men with SCI	5	In 97% of men with SCI, penile vibratory stimulation (PVS) and/or electroejaculation (EEJ) were indicated to obtain an ejaculate. The motility of sperm plays an important role in intrauterine or intravaginal insemination. Also, surgically retrieved sperm from the testis or the epididymis is used for in vitro fertilization with intracytoplasmic sperm injection.
Čehić et al. [44]	Narrative Literature Review	n/a	3 to 4 (acting as a narrative review of clinical data)	In men with SCI, erectile dysfunction is treated by using phosphodiesterase-5 inhibitors, intracavernosal injections, vacuum devices, and penile prostheses. Penile vibratory stimulation (PVS), electroejaculation (EEJ), prostate massage, or surgical procedures are used to obtain semen. Pregnancy rates were comparable with those of able-bodied subfertile cohorts.
Ibrahim et al. [45]	Narrative Literature Review Article	n/a	4 to 5 (depending on the specific grading system)	In men with SCI, penile vibratory stimulation (PVS) and electroejaculation (EEJ) are the primary methods to collect sperm. Τhe risk of developing autonomic dysreflexia should be taken into account prior to administering any ejaculation approach, particularly in patients with T6 and rostral levels of injury.
Ibrahim et al. [46]	Narrative Literature Review Article	n/a	4	In men with SCI, erectile and ejaculatory dysfunction, along with abnormal semen quality, are the primary complications. Most of the patients had low sperm motility and viability. Given that the total amount of sperm retrieved showed good motility, intravaginal or intrauterine insemination techniques may be recommended for fatherhood.
Fode M, et al. [47]	Clinical Narrative Review Article	n/a	4	In men with SCI, neurogenic anejaculation occurs, and penile vibratory stimulation (PVS) or electroejaculation (EEJ) are usually recommended for semen retrieval. Aspira­tion from the vas deferens or the epididymis, as well as testicular biopsy or surgery, constitutes other methods to collect sperm.
Gat et al. [48]	Retro­spective Comparative Clinical Study	15 men with psychogenic anejaculation, and 22 men with SCI	3	Electroejaculation (EEJ) combined with intracytoplasmic sperm injection (ICSI) may be recommended for couples with psychogenic anejaculation. Chances for pregnancy in psychogenic anejaculation seem to be comparable to those in SCI patients.
Sønksen et al. [49]	Retro­spective Cohort Study	140 men with SCI and anejaculation, along with their partners	4	Penile vibratory stimulation (PVS) associated with vaginal self-insemination is proposed for assisted conception in couples in whom, although the male partner presents with SCI, there are no sperm motility problems while the female partner is fine.
O'Kelly et al. [50]	Retro­spective Historical Review	78 men with SCI	4	In men with SCI, anejaculation represents the absence of seminal emission in the posterior urethra. Also, anejaculation in men with SCI was found to be accompanied by retroperitoneal lymph node dissection as well as by sacral autonomic disruption.
McGuire et al. [51]	Retro­spective Review Study	31 men with SCI	4	In men with SCI presenting anejaculation, electroejaculatory stimulation can be used to provide semen for pregnancy via assisted conception, if required.
Iremashvili et al. [52]	Retro­spective Analysis	41 men with SCI	4	In azoospermic men with SCI, the issue of the assisted ejaculation procedure is discussed. It seems that in these patients, the electroejaculation (EEJ) is preferred over penile vibratory stimulation (PVS), prior to surgical sperm retrieval.
Dimitriadis et al. [53]	Narrative Literature Review	n/a	5	In men with SCI, the completeness of the injury and the level of neurological damage assess the severity of sexual dysfunction. To restore the decreased quality of the semen, penile vibratory stimulation (PVS) or electroejaculation (EEJ) is recommended. The assessment of sperm motility is also of major importance for the successful implementation of assisted conception.
Brackett et al. [54]	Retro­spective Clinical Review	500 men with SCI	4	In 86% of men with SCI, who had a T10 or rostral injury level, penile vibratory stimulation (PVS) was used as the method of choice for semen retrieval in order to achieve assisted fertilization. Electroejaculation was also alternatively used when the PVS method failed.
Biering-Sørensen et al. [55]	Clinical Observational Study/Case Series	14 men with SCI	4	In men with SCI, the importance of the penile vibratory stimulation (PVS) method to manage infertility, spasticity, and neurogenic detrusor overactivity has been studied. Α percentage greater than 80% of the participating patients was found to be able to ejaculate via PVS.

**Table 4:** Main findings and characteristics of the studies, based on autonomic dysreflexia, assisted reproductive technologies (ART), surgical interventions and clinical barriers, and structural approaches in men with SCI SCI: spinal cord injury

Study	Study design	Sample size (participants)	Level of evidence	Main findings and characteristics
Autonomic Dysreflexia (AD)				
Courtois et al. [56]	Systematic Review	n/a (37 different published papers and research studies were included)	2b or 3 (as it draws conclusions from previously published observational, clinical, and experimental studies rather than a single Randomized Controlled Trial)	In men with SCI, ejaculation may be a cause of autonomic dysreflexia (AD). Nifedipine, prazosin, captopril, and clonidine are medications included in the specific treatment for AD triggered by sexual activities in these patients. Nifedipine appears to be the most significant treatment for AD, either acutely or preventively.
Trueblood et al. [57]	Narrative Literature Review Article	n/a	5	Autonomic dysreflexia occurs mainly in high-level SCI and induces the appearance of cardiovascular symptoms. In these cases, neurological control of the cardiovascular system is disrupted, while at the same time the function of the renin-angiotensin system (RAS) is dysregulated. The proposed treatment is cell transplantation with selective inhibition of RAS.
Alwashmi [58]	Case Report	1 man with SCI	4	Emergence of autonomic dysreflexia in SCI patients is particularly presented when the injury is located at the sixth thoracic vertebra (T6) and above. A significant cause of autonomic dysreflexia in spinal cord injuries appears to be the distention of the bladder that can occur in these conditions.
Bilgin Badur et al. [59]	Book chapter	n/a	n/a	Up to 90% of patients with cervical or high-thoracic spinal cord injury are susceptible to the emergence of AD. Also, the initial presenting symptom is an acute, severe headache.
Assisted Reproductive Technologies (ART)				
Engin-Uml Stün et al. [60]	Retrospective, non-Randomized Comparative Study.	44 men with SCI	2b	In men with SCI, pregnancy outcomes of using sperm retrieval methods such as prostatic massage, electroejaculation (EEJ), or testicular sperm extraction (TESE) were found to be comparable.
Čehić et al. [61]	Retrospective Cohort Study	82 men with SCI, and 74 men with obstructive azoospermia (OA)	2b	It has been found that cryopreserved sperm collected by using the testicular sperm aspiration technique (TESA) can be proposed for assisted reproduction via intracytoplasmic sperm injection (ICSI) in order to manage infertility attributed to azoospermia due to SCI.
Reignier et al. [62]	Retrospective Cohort Study.	78 men with SCI	2b to 3 (typically categorized as a Retrospective Cohort or Case-control Study).	Assisted reproductive technology (ART) with the use of frozen-thawed spermatozoa ensures similar results in men with SCI compared to the infertile population. Penile vibratory stimulation (PVS), electroejaculation (EEJ), and testis biopsies were used for sperm retrieval.
Kathiresan et al. [63]	Retrospective Analysis	82 couples (male infertility due to SCI)	2b	In couples where men had SCI, intravaginal (IVI) or intrauterine insemination (IUI) methods appear to be acceptable for pregnancy by using assisted technology (ART).
Leduc BE [64]	Retrospective Study	31 couples with spinal-cord-injured (SCI) male partners	4	Intravaginal (IVI) and intrauterine insemination (IUI) or in vitro fertilization were used to treat infertility in men with SCI. A pregnancy rate of 55% was achieved.
Kathiresan et al. [65]	Retrospective Comparative Study	31 couples with men suffering from SCI, and 297 couples without SCI in male partners	3	In men with SCI, in vitro fertilization (IVF) and intracytoplasmic sperm injection (ICSI) showed lower fertilization rates, although identical results for pregnancy and live births were found between men with or without SCI.
Brackett et al. [66]	Clinical Review	500 men with SCI	2b to 3 (based on Cohort Studies and Case series)	Penile vibratory stimulation (PVS), electroejaculation (EEJ), prostate massage, or surgical sperm retrieval are recommended to collect sperm in men with infertility due to SCI. Low sperm motility and semen abnormalities enhance the problem. Assisted reproductive techniques (ART) are used for fatherhood.
Surgical Interventions				
Pang et al. [67]	Systematic Review	475 men with SCI	3	In men with SCI, penile prosthesis (PP) insertion constitutes an effective option to treat end-stage erectile dysfunction (ED) or urinary function. However, there are risks for erosion, infection, or implant explantation. Inflatable PP seems to be the first treatment option for these patients.
Kim et al. [68]	Retrospective Cohort Study and Clinical Review.	48 men with SCI	4	Malleable penile prosthesis insertion in men with erectile dysfunction due to SCI provides high satisfaction and low complication rates.
Zermann et al. [69]	Retrospective, Observational Case Series.	245 neurologically impaired men (of whom 197 had SCI)	4	In men with SCI, penile prosthesis implantation seems to be useful in order to manage erectile dysfunction and/or urinary incontinence. And 122 (90.3%) and 76 patients (82.6%), respectively, with urinary and erectile dysfunction were successfully managed. Penile prosthesis appears to be safe in patients with this type of neurological impairment.
Sievert et al. [70]	Prospective Observational Study	10 men with SCI	4	Early implantation of bilateral sacral nerve modulators (SNMs) in patients with complete SCI seems to improve lower urinary tract dysfunction, prevent detrusor overactivity and urinary incontinence, provide normal bladder capacity, reduce infections in the urinary tract, and enhance bowel and erectile function, while the nerves remain intact.
Lombardi et al. [71]	Retrospective, non-blinded Clinical Study without controls.	75 participants with SCI	4	Sacral neuromodulation has been found to be useful in patients presenting with incomplete SCI, particularly in the case of neurogenic bowel symptoms (NBSs) and neurogenic lower urinary tract symptoms (NLUTSs). Short Form 36 Health Survey questionnaire (SF-36) was used to examine quality of life (0.01), with all patients improving their scores by 20% compared to the baseline.
Clinical Barriers and Structural Approaches				
Aikman et al. [72]	Scoping Review	n/a (20 research articles were included)	5	In men with SCI, physiological modifications and psychological and social factors affect sexuality. Lack of sexual rehabilitation services and consensus regarding clinician roles, as well as societal stigma related to sexual disability, are all considered to be barriers to the recovery of sexual function in these patients.
Akman et al. [73]	Cross-Sectional Study	47 men with SCI	4	In men with SCI, erectile function was assessed by using the International Index of Erectile Function-5 (IIEF-5) questionnaire. Sexual activity following injury was found to be of major importance for quality of life, while the role of sexual education was also underlined for these patients.

It should also be reported that, as this manuscript is a narrative review, no quantitative meta-analysis was performed. Effect sizes reported are those from the original studies cited.

Results

Neurological Severity and Functional Outcomes

Gao et al. demonstrated a strong correlation between SCI severity and recovery of sexual function [12]. In patients with incomplete SCI involving thoracic and lumbar segments, erectile function significantly declined over a two-year follow-up period, often accompanied by premature ejaculation. The degree of erectile impairment was directly associated with the extent of spinal cord damage and the neurological level of injury. Higher-level injuries and more extensive lesions were consistently linked to poorer recovery outcomes. Moreover, the timing of surgical intervention was shown to be critical. Delayed decompression was associated with increased rates of persistent ejaculatory dysfunction, whereas early surgical management significantly improved recovery trajectories. Approximately 70% of patients with incomplete SCI achieved satisfactory restoration of erectile function within two years, highlighting the importance of injury completeness in prognostication [12].

The level and completeness of SCI are primary determinants of sexual function outcomes. Lesions above T10 generally preserve reflex erections mediated by sacral parasympathetic pathways but impair psychogenic erections due to disruption of descending sympathetic pathways. In contrast, lesions affecting lower spinal segments impair reflexogenic erections while potentially preserving psychogenic responses, although often insufficient for satisfactory sexual performance. Sacral and cauda equina lesions severely disrupt reflex arcs, leading to profound erectile and ejaculatory dysfunction. The evaluation of sacral reflexes, including the bulbocavernosus and anal reflexes, is essential for assessing residual sexual function and guiding therapeutic strategies [4,5]. In addition, according to more recent studies, it seems that the level and the completeness of injury strongly influence the sexual potential, affecting both the erectile function and the ejaculation. Complete lesions cause greater impairment, while incomplete lesions, with preserved sacral reflexes, are associated with more frequent erections and ejaculations [13].

Neurophysiological Mechanisms

Neurophysiological studies provide further insight into the mechanisms underlying sexual dysfunction following SCI [14-16]. Chéhensse et al. reported a significant reduction in the number of rhythmic contractions of perineal muscles in lesions below the L3 level, with complete absence of contractions in injuries involving sacral nerve roots. These findings may reflect impairment of sacral reflex arcs critical for ejaculatory function [14]. Electrophysiological investigations reinforce these observations. In a cohort study involving 98 men with SCI and sexual dysfunction, the prevalence of ejaculatory disorders was markedly higher compared to controls (73.7% vs. 19.7%). Electromyographic assessment revealed increased polyphasic potentials suggestive of denervation, along with absence of the bulbocavernosus reflex in a substantial subset of patients. Additionally, prolonged latency in sensory-evoked potentials and abnormalities in sympathetic skin responses were observed, findings that may indicate impairment of both afferent and efferent neural pathways. Elevated somatosensory thresholds following dorsal penile nerve stimulation further confirmed reduced neural conduction and sensory integration, although causality cannot be established from their observations alone [15].

In another study, the presence of the bulbocavernosus reflex after spinal cord injuries above the T12 level was correlated with the occurrence of reflexogenic erection. Conversely, the absence of reflexogenic erection tended to coincide with the simultaneous absence of the bulbocavernosus reflex. Furthermore, the presence of perineal sympathetic skin responses (pSSRs) was correlated with the occurrence of psychogenic erection, and vice versa [16]. These findings support a potential relationship between preserved neurophysiological responses and sexual function after SCI; however, considerable interindividual variability exists, and the underlying mechanisms are likely multifactorial.

Quality of Life and Psychosocial Dimensions

Sexual dysfunction has a substantial negative impact on QoL in men with SCI, through influencing psychological, relational, and social factors [13,17,18]. Depression, anxiety, negative self-perceptions, and impaired body image have been associated with poorer sexual functioning, including reduced sexual identity, arousal, and activity [13]. Studies using validated instruments, such as the Life-Satisfaction Questionnaire-9 (LiSat-9) and General Health Questionnaire (GHQ-12), consistently demonstrate significantly lower QoL scores compared to pre-injury levels and the general population [17,18]. Nearly half of patients report low QoL in conjunction with erectile dysfunction and hypogonadism [17]. Severe erectile dysfunction is observed in up to 27% of individuals, accompanied by lower quality of life (QoL) scores [18]. The prevalence of sexual dysfunction among married men with SCI reaches 67.2%, underscoring its widespread impact [19].

Psychological adaptation, partner dynamics, and relationship satisfaction are critical determinants of sexual outcomes, associated with better sexual adjustment and overall sexual well-being following SCI [13,19,20]. Improved couple adjustment is associated with reduced emotional distress, decreased embarrassment, and enhanced sexual satisfaction [19,20]. Importantly, sexual desire often remains stable or may even increase over time, despite persistent functional limitations. Barriers to sexual activity include social stigma, environmental limitations, lack of accessible spaces, and comparison with able-bodied individuals. Mood disorders, including depression and anxiety, further contribute to reduced sexual desire, whereas physical activity may improve sexual engagement [20]. Furthermore, lifestyle modifications, including dietary adjustments (with careful regulation of protein, carbohydrate, and fat intake, as well as caloric restriction) and physical exercise, have been proposed as potential adjunctive strategies, although current evidence remains limited [21].

The findings underline the importance of patient-centered sexual rehabilitation programs that extend beyond the management of physiological dysfunction, incorporating psychological support, partner involvement, sexual counseling, and relationship-centered interventions. 

Semen Parameters and Fertility Potential

Semen quality is consistently impaired in men with SCI, representing a major contributor to infertility [22-29]. In a cohort of 35 patients, sperm concentration remained within normal limits; however, significant reductions in motility and vitality were observed, accompanied by increased morphological abnormalities. Elevated levels of inflammatory markers, granulocytes, and oxidative stress were also reported, suggesting a chronic inflammatory milieu contributing to sperm dysfunction [22]. Comparative studies between SCI patients and men with idiopathic infertility revealed distinct differences in semen characteristics. Although semen volume was significantly lower in SCI patients (1.5 mL vs. 3.1 mL), sperm concentration and total sperm count were paradoxically higher. Despite this, progressive motility was markedly reduced (5.0% vs. 35.0%), leading to significantly lower fertilization rates (50% vs. 75%). These findings indicate that qualitative sperm abnormalities may be presented as more significant contributors to infertility in men with SCI than quantitative sperm parameters [23].

Further analyses demonstrated increased sperm DNA fragmentation (SDF), elevated reactive oxygen species (ROS), and higher rates of chromosomal aneuploidy in SCI patients. Leukocytospermia and increased pro-oxidant capacity were also observed, reinforcing the role of oxidative stress in sperm damage. Notably, patients with complete SCI (ASIA A) exhibited significantly lower sperm motility compared to those with incomplete injuries (ASIA B-D), suggesting a potential association between injury severity and sperm function. Additionally, higher sperm concentrations were reported in patients with cervical injuries compared to those with thoracic lesions, indicating potential regional differences in testicular function or seminal emission [24]. Zinc levels in semen, a critical factor in spermatogenesis and antioxidant defense, were significantly reduced in SCI patients (85.20 mg/L vs. 147.16 mg/L in controls). This deficiency may contribute to impaired sperm motility and increased susceptibility to oxidative damage [25].

Interpretation of semen-quality findings across studies should be undertaken with caution due to substantial methodological heterogeneity. Semen parameters may be influenced by factors including abstinence duration, semen collection method, time elapsed since injury, and severity of injury. Nevertheless, despite these methodological differences, reduced sperm motility and vitality represent the most consistent findings in men with SCI across studies.

Mechanisms of Ejaculatory Dysfunction

Spinal cord injury disrupts both the emission and expulsion phases of ejaculation through impairment of autonomic and somatic pathways [30,31]. Dyssynergia between the bladder neck and the external urethral sphincter frequently results in incomplete semen expulsion and/or retrograde ejaculation. Semen may remain within the prostatic urethra, further compromising fertility potential. Lesions affecting the lumbar and sacral spinal segments are particularly associated with premature, involuntary, or absent ejaculation. These findings highlight the critical role of intact sacral reflexes and coordinated autonomic function in normal ejaculatory physiology. Additionally, retrograde ejaculation may often go unrecognized, necessitating careful evaluation during clinical assessment of fertility [31].

Therapeutic Interventions

*Pharmacological management*: Phosphodiesterase type-5 (PDE-5) inhibitors remain the first-line therapy for erectile dysfunction in men with SCI [32-38]. A meta-analysis of randomized controlled trials involving 1,492 patients demonstrated that these agents are approximately four times more effective than placebo [32]. Among available agents, tadalafil was associated with higher efficacy estimates, followed by vardenafil and sildenafil, although comparative evidence remains limited [32,35]. The prolonged duration of action of tadalafil allows for greater spontaneity in sexual activity, which may improve patient satisfaction [35]. However, treatment response varies depending on the neurological level of injury. Reduced efficacy has been reported in lesions below T10, likely due to disruption of relevant neural pathways required for pharmacological response. Treatment responsiveness is heterogeneous and may be influenced by parameters such as neurological level and completeness of injury, vascular integrity, and individual patient characteristics [36].

Common adverse effects include headache, nasal congestion, and myalgia, while serious complications such as priapism and hypotension are rare but clinically significant. As in the general population, PDE-5 inhibitors are contraindicated in patients receiving nitrate therapy and should be cautiously administered in patients with symptomatic hypotension [32,35].

The first-line pharmacological treatment for premature ejaculation includes the daily administration of selective serotonin reuptake inhibitors (SSRIs), as they maintain elevated serotonin levels, which in turn suppress ejaculation [3].

In conclusion, PDE-5 inhibitors are considered the first-line treatment for the treatment of erectile dysfunction in men with SCI, while vacuum erection devices and the placement of a penile prosthesis are the second and third choices, respectively, for this specific therapeutic treatment.

*Assisted ejaculation techniques*: Penile vibratory stimulation (PVS) and electroejaculation (EEJ) are key modalities for semen retrieval in men with SCI [39-56]. PVS is particularly effective in patients with lesions above T10 and intact sacral reflexes, achieving ejaculation in up to 86% of cases. In cases of initial failure, the use of dual vibratory devices can increase success rates to approximately 80% [39]. Additionally, in another research study involving 140 couples of male patients with anejaculation due to spinal cord injury and their healthy partners, PVS and intravaginal insemination (IVI) using a syringe were conducted. It was observed that among 60 of the 140 couples, a total of 82 pregnancies were recorded, corresponding to a pregnancy rate of 42.8% across the entire cohort of 140 couples [49]. EEJ represents a highly effective alternative, particularly in patients with lower-level lesions or unsuccessful PVS attempts [40-48, 50-55]. Success rates of up to 98. 7% have been reported. Repeated EEJ procedures have been shown to improve sperm motility outcomes in patients with initially poor-quality samples [42].

However, caution is required in patients with lesions above T6 due to the risk of autonomic dysreflexia during ejaculation, assisted or non-assisted, necessitating prophylactic management [56]. Also, autonomic dysreflexia, which negatively affects the cardiovascular system, is often attributed to high-level spinal cord injuries and is characterized by episodes of high blood pressure combined with bradycardia, which is life-threatening. Moreover, due to spinal cord injury, the nervous system's control over cardiovascular function is interrupted, resulting in dysregulation of the renin-angiotensin system, which is normally involved in the regulation of arterial blood pressure. It appears that cell transplantation, such as fetal parasympathetic neurons derived from the brain stem or specific raphe regions, into the injured spinal cord, restores the regulation required by the sympathetic nervous system for proper cardiovascular function [57]. Bladder distension is the primary cause of autonomic dysreflexia’s occurrence [58]. Clinical manifestations presented in this disorder are severe headache, bradycardia, facial flushing, pallor, cold skin, as well as sweating in the lower part of the body. It should also be emphasized that early diagnosis of autonomic dysreflexia and treatment, often by irrigating or changing the Foley catheter, can prove to be life-saving for the patient [59].

In summary, concerning assisted ejaculation techniques, PVS is generally considered the preferred first-line sperm retrieval method. The advantages of PVS use in men with SCI are that the method is non-invasive, is relatively inexpensive, and is associated with high success rates in men with lesions above the T10 neurological level of injury. Moreover, EEJ is typically applied in men with SCI presenting unsuccessful sperm retrieval via PVS, as well as in men with SCI in whom the level of injury is more caudal than T10. Nevertheless, the use of EEJ shows a higher risk of impending autonomic dysreflexia compared to the PVS method. As for the surgical sperm retrieval techniques, they should be considered when non-surgical approaches fail.

*Assisted reproductive technologies*: Assisted reproductive technologies (ART), including intracytoplasmic sperm injection (ICSI), intravaginal insemination (IVI), and intrauterine insemination (IUI), play a central role in achieving fertility in men with SCI [60-66]. Pregnancy rates achieved through ART may be comparable to those observed in other causes of male infertility, although comparisons are limited by study heterogeneity [61,65]. Studies demonstrate that the method of sperm retrieval (PVS, EEJ, or surgical extraction) does not significantly affect pregnancy outcomes, although these findings should be interpreted cautiously because of differences in patient selection, sperm quality, and assisted reproductive protocols [60,61,65]. In clinical practice, IVI and IUI offer less invasive options, with pregnancy rates of 37.8% and 24.6%, respectively [63].

It should be pointed out that interpretation of fertility outcomes across studies should be treated with caution, as pregnancy rates are influenced by multiple confounding factors, including female partner fertility status, semen preparation protocols, injury level, sperm quality, and the use of adjunctive assisted reproductive techniques.

The choice of IVI, IUI, and ICSI methods is typically individualized based on parameters such as semen quality, female reproductive factors, previous treatment outcomes, and resource availability. Less invasive techniques such as IVI and IUI are low cost and widely available, but they present lower success rates compared to ICSI, which, although invasive and more expensive, has higher fertilization rates.

*Surgical interventions*: Penile prosthesis implantation is an effective option for patients with severe erectile dysfunction due to SCI [67-69]. Satisfaction rates range from 79.2% to 92.9%, with a substantial proportion of patients reporting successful sexual intercourse [67]. However, complication rates are higher compared to non-SCI populations, including infection, erosion, and device removal due to dissatisfaction [67,68].

Sacral neuromodulation (SNM) represents an emerging therapeutic approach with potential benefits extending beyond urinary function. Studies have demonstrated improvements in erectile function, bowel and bladder control, and overall QoL [70,71]. Significant increases in IIEF-5 scores have been reported following SNM implantation, with some patients achieving sexual function without pharmacological support [71].

Clinical Barriers and Structured Approaches

Sexual health is a key concern for many male patients with SCI but is often inadequately addressed by healthcare providers. Major barriers include limited knowledge, lack of training, insufficient experience, and poor interdisciplinary collaboration. Clinicians frequently expect patients to initiate discussions, while patients may feel embarrassed or uncertain about where to seek help [72,73]. The P-LI-SS-IT model provides a structured framework for addressing sexual health concerns. It consists of four levels: Permission, Limited Information, Specific Suggestions, and Intensive Therapy. Permission encourages open communication between clinician and patient. Limited information focuses on providing targeted education about the specific dysfunction. Specific Suggestions involve individualized management strategies. Intensive Therapy includes referral to specialized professionals. Familiarity with available specialized services is essential for appropriate referral and comprehensive care [73].

## Conclusions

Sexual dysfunction and infertility following SCI are complex and multidimensional conditions involving neurological, physiological, biochemical, and psychosocial components. Current evidence suggests that semen quality impairment is largely driven by oxidative stress and inflammatory processes, and ejaculatory dysfunction appears to be primarily associated with disruption of neural pathways.

Despite these challenges, advances in pharmacological therapies, assisted ejaculation techniques, and reproductive technologies may improve sexual and reproductive outcomes in men with SCI. Nevertheless, the reproductive outcome response is heterogeneous and depends on factors such as neurological level, completeness of injury, semen quality, and female reproductive factors. These findings underscore the importance of a comprehensive, multidisciplinary approach to management, tailored to the individual patient's neurological status and reproductive goals.

The findings of this narrative review should be interpreted in light of the study's methodological limitations. Further studies are needed to clarify underlying mechanisms and optimize therapeutic strategies in men with sexual dysfunction and infertility following SCI.
